# Laser Treatment in Oral Cavity Soft Tissue in Pediatrics; Current Knowledge and Future Directions

**DOI:** 10.31661/gmj.v13iSP1.3683

**Published:** 2024-12-08

**Authors:** Hossein Shahoon, Ali Rafighi, Mehdi Ashrafi, Hossein Ebrahimi, Sedighe Mozafar

**Affiliations:** ^1^ Oral and Maxillofacial Surgery Department, Faculty of Dentistry, Shahed University, Tehran, Iran; ^2^ Department of Orthodontics, Dentistry Faculty, Tabriz University of Medical Sciences, Tabriz, Iran; ^3^ Department of Periodontics, Faculty of Dentistry, Shahed University, Tehran, Iran; ^4^ Department of Pediatric Dentistry, Shahed University, Tehran, Iran

**Keywords:** Laser, Oral, Soft Tissue, Children

## Abstract

This review explores the advancements in laser therapy for pediatric oral cavity soft tissue problems, highlighting its therapeutic implications and advantages over conventional treatments. The research began with a comprehensive literature search in December 2023, identifying 159 articles related to laser treatments in pediatric dentistry. After eliminating duplicates, 84 distinct articles were analyzed, leading to the selection of 5 relevant studies. The findings indicate that low-level laser therapy (LLLT) offers significant benefits, including enhanced wound healing, pain relief, and reduced postoperative complications such as bleeding and infection. Various laser types, including Er:YAG, CO2, and diode lasers, have shown effective clinical outcomes in procedures like frenectomy and gingival re-contouring. Notably, LLLT’s non-invasive nature minimizes edema and inflammation, making it particularly suitable for young patients who may be anxious about dental procedures. The review emphasizes that lasers can be utilized as either primary or supplementary tools in both surgical and non-surgical interventions, leading to improved patient experiences and outcomes. Furthermore, the paper discusses the importance of understanding the physical principles of laser therapy, as different wavelengths interact uniquely with various tissues, influencing treatment efficacy. Overall, this review advocates for the judicious application of laser technology in pediatric dentistry, aiming to enhance treatment outcomes and patient satisfaction while addressing the challenges associated with traditional methods.

## Introduction

The acronym LASER signifies light amplification through stimulated emission of radiation, a term first articulated in a scholarly paper published in 1959 by graduate student Gordon Gould from Columbia University. In 1917, the physicist Albert Einstein elucidated the theory of stimulated emission, thereby unveiling the foundational principles underlying laser technology [1][2]. The inaugural efficient laser was engineered by Theodore Meimann at the Hughes Research Laboratory in 1960 [3]. The CO2 laser was conceived in 1964 by Patel at Bell Laboratories; however, its application in oral surgery for the excision of soft tissue lesions did not emerge until the 1980s. A laser specifically designed for dental applications received approval from the Food and Drug Administration in 1990. Presently, it is extensively employed in treating the hard tissues of teeth. The hard tissue laser presents a viable alternative to traditional drilling methods, fostering an improved clinical environment for dental practitioners, which subsequently enhances treatment outcomes and patient satisfaction [3][4]. Furthermore, the use of sharp dental instruments, the audible noise of drilling, and associated vibrations can be mitigated during dental procedures, offering significant advantages, particularly in pediatric dentistry. Recent advancements in laser technology have enabled its effective utilization for the diagnosis, prevention, and treatment of dental caries, in addition to facilitating minimally invasive procedures. The American Academy of Pediatric Dentistry advocates the judicious application of both soft tissue and hard tissue lasers for a variety of oral procedures in infants, children, and adolescents [2]. The applications of lasers within pediatric dentistry serve as an alternative modality that may, at times, complement or supersede conventional methodologies: diverse applications on both soft and hard tissues are achievable through the utilization of various laser wavelengths. It is crucial to acknowledge that the underlying physical principles governing laser therapy must not be overlooked concerning other scholarly works and publications; different wavelengths exhibit distinct interactions with the various chromophores—such as hemoglobin, water, and hydroxyapatite—present within the target tissues, including mucosa, gums, and tooth structures [5][6]. Consequently, the efficacy of treatment is contingent upon the optical affinity and distinct absorption coefficients of each specific tissue type relative to the respective wavelength. The absorption coefficient for water is denoted as (nm) and is measured at 860 for the CO2 laser (carbon dioxide laser) operating at a wavelength of 10,600 nm. After the invention of the diode laser, the utilization of laser technology within the domain of dentistry became increasingly prevalent [3][7].

Lasers in dentistry are highly tolerable and well-accepted by children, which can enhance treatment outcomes and facilitate both surgery and recovery. The benefits include minimal or no post-operative complications such as bleeding, scarring, and infection; effective homeostasis due to the thermocoagulation effect; the elimination of the need for sutures; a lack of pain or pain levels comparable to traditional surgical procedures; and a reduction in operation time, all of which contribute to the advantages of laser treatment. The purpose of this review is to investigate the various laser therapies in pediatric oral cavity soft tissue problems and their therapeutic implications to overcome resistance to conventional treatments.

## Literature Search and Selection of Articles

**Figure-1:**
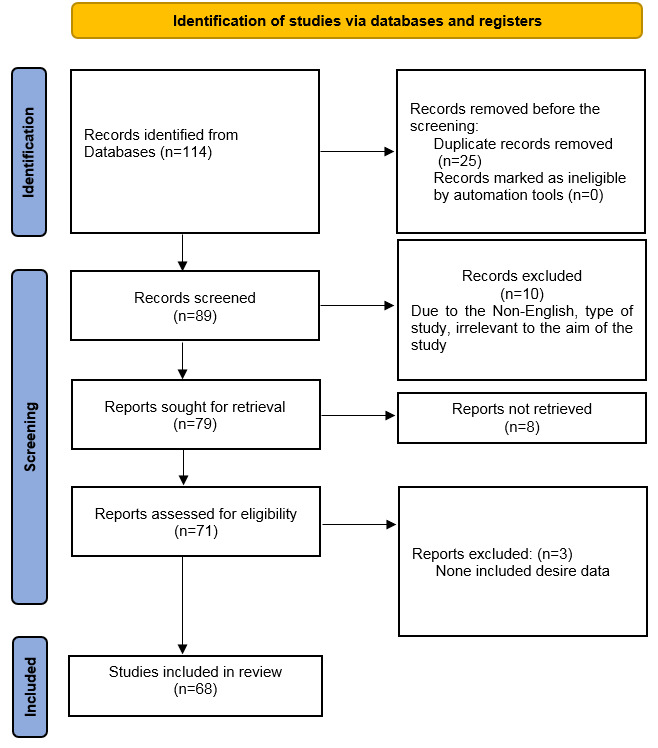
Figure 1. Flow diagram of the steps for including studies in the review study

An extensive review of the current literature on recent advancements in laser therapy on dental soft tissues problems in pediatrics was undertaken. The inclusion criteria encompassed articles written in English, available in full-text, comprehensive, and directly pertinent to the subject under investigation. A comprehensive search was carried out in the PubMed and Scopus databases in December 2023, utilizing keywords related to laser, laser treatment, oral cavity soft tissue, pediatric oral soft tissue, and novel therapeutic methods. Initially, 159 articles were identified based on their titles, abstracts, and publication dates. After eliminating duplicate entries, 84 distinct articles were retained. These articles were thoroughly analyzed, and a subset of 5 articles relevant to the research question were selected. Subsequently, in March 2024, a supplementary search was conducted using Google Scholar, PubMed, and Scopus, identifying and including nine additional articles directly related to the topic of interest (Figure-1).

## Classification of Laser

The laser emission constitutes a single wavelength monochromatic light that is generated through the stimulation of a synthetic material. For the purposes of incising, cutting, and ablating, it employs light energy characterized by a continuous and uniform emission from the light chamber to the targeted tissue. An active medium, which can be a gas, crystal, solid state, or semiconductor, is stimulated to produce photons of energy, with lasers frequently being designated by their respective active mediums. The distinct wavelength associated with different lasers is a critical determinant of their clinical applications. Chromophores or light-absorbing pigments are intrinsic to oral and dental hard tissues, wherein they facilitate the absorption of laser energy at specified wavelengths [8].

Typically, biostimulation protocols are characterized by the utilization of lasers classified as class I, II, and III, which are distinguished by their power output of less than 1 W, and are conventionally referred to as low-level laser therapy (LLLT) due to their relatively minimal energy emission. In stark contrast, the devices categorized as class IV laser systems possess an intensity that exceeds 1 W, thereby earning their designation as high power laser therapy (HPLT), which implies a significant increase in energy delivery for therapeutic purposes [9]. The guidelines established by the Multinational Association of Supportive Care in Cancer (MASCC) and the International Society of Oral Oncology (ISOO) have provided robust support for the assertion that intraoral photobiomodulation (PBM) utilizing LLLT plays a crucial protective role in the prevention of oral mucositis (OM) among adult patients who are undergoing conditioning regimens for hematopoietic stem cell transplantation (HSCT), whether or not they are also receiving total body radiation (TBI). Likewise, a similar protective effect has been observed in patients diagnosed with head and neck cancers who are undergoing radiotherapy (RT) as well as those who receive a combination of RT and chemotherapy (CT), which highlights the versatility and effectiveness of LLLT in various clinical contexts[10]. Drawing upon the encouraging data that has been reported in the adult demographic, the Paediatric Oncology Group of Ontario (POGO) has advocated for the implementation of LLLT as a preventive measure in pediatric patients who are undergoing chemotherapy or are engaged in pretransplant conditioning regimens that are associated with a notably high incidence of mucositis, thereby emphasizing the importance of this therapeutic modality across different age groups. Consequently, the collective findings underscore the necessity of further research and application of LLLT, aiming to establish it as a standard practice in the management of oral complications arising from oncological treatments [9][10][11].

## Laser‑tissue Interaction

Upon the application of laser light to target tissue, a photothermal reaction is initiated, resulting in heat generation and an elevation of temperature within the tissue. When this temperature exceeds 60°C, it induces protein coagulation within the tissue. Conversely, when the temperature surpasses 100°C, it leads to the vaporization of water molecules and the ablation of soft tissue. However, a temperature exceeding 200°C is requisite for procedures involving hard tissue. Upon the interaction of laser light with target tissue, four distinct types of interactions occur, contingent upon the optical properties of the target tissue and the wavelength of the laser light. These interactions are elucidated as follows [12]:

• Absorption of laser light

• Transmission of laser light

• Reflection of laser light

• Scattering of laser light

Absorption

The phenomenon of absorption, which is fundamentally associated with the presence of chromophores within the target tissue, plays a critical role in the interaction between laser light and biological tissues, as it is this specific chromophore that facilitates the effective absorption of the laser light by the tissues in question. Numerous studies have shown that various wavelengths of laser light exhibit distinct coefficients of absorption when interacting with the different components present in hard and soft tissues, which include essential elements such as mineral content, water, blood components, and pigments that are intrinsic to the tissues. Specifically, laser light that possesses a shorter wavelength, particularly in the range of 500 to 1000 nanometers, is predominantly absorbed by the components of blood and pigments found within the tissues, whereas, conversely, laser light with longer wavelengths tends to have a significantly higher affinity for hydroxyapatite crystals and water molecules, which are crucial constituents of the hard tissue matrix [12][13][14].

Transmission

The transmission of laser light through the target tissue can occur without inducing any biological effect, a property that is largely contingent upon the specific wavelength of the laser light being utilized in the procedure, highlighting the importance of selecting the appropriate wavelength for desired outcomes. For instance, lasers belonging to the erbium family, alongside the CO2 laser, are known for their efficient absorption by tissue fluids, thereby resulting in minimal transmission through these fluids, whereas, in contrast, the laser energy emitted from argon and Nd-YAG lasers tends to be transmitted into the adjacent tissue when it interacts with tissue fluids, which can lead to unintended consequences depending on the context of the treatment being administered.

Reflection

The reflection of laser light from the target tissue can occur without causing any discernible effect on the tissue itself, yet this unintentional reflection poses a potential risk, particularly to the eyes of the clinician or any individuals present in the vicinity of the procedure. It is important to note that while this reflective property can be hazardous, it is also strategically utilized by caries-detecting lasers, which leverage this capability to assess and measure the integrity of sound tooth structures, thereby providing valuable diagnostic information in clinical practice [12][13][14].

Scattering of laser light

The scattering of laser light leads to the transfer of heat and can contribute to the damage of tissues that are adjacent to the primary target area, a phenomenon that is critical to consider as it may significantly diminish promising clinical outcomes in various medical interventions. Nonetheless, this scattering property can be advantageous in certain clinical scenarios, particularly when the clinician aims to treat conditions such as aphthous ulcers or when performing procedures that involve the curing of composite resin restorations, as it allows for a more comprehensive treatment approach that can enhance the overall efficacy of the therapeutic intervention [12][13][14].

## Different Lasers and their Applications in the Pediatrics Dental Field

**Figure-2:**
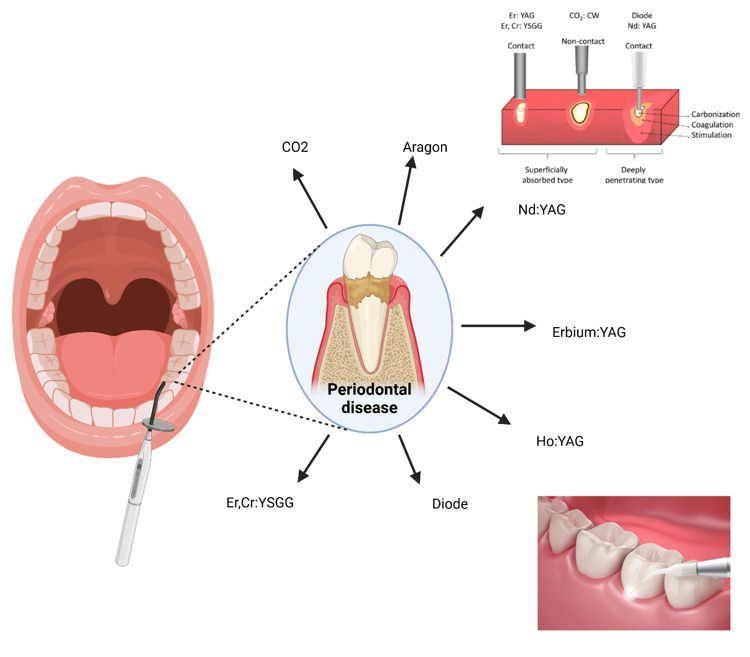
Figure 2. Schematice type of lasers in dental treatment

**Table:** Table1. Characteristics of Dental Laser [15][16][17][18]

**Laser Type**	**Wavelength**	**Mode**	**Application**
CO_2_	10 600 nm	Pulse or continuous-wave	1. Ablation of soft tissues 2. Aesthetic gingival contouring 3. Management of oral ulcerative lesions 4. Frenectomy and gingivectomy procedures 5. Removal of necrotic epithelial tissue in regenerative periodontal surgeries
Nd:YAG (neodymium-doped yttrium aluminum garnet)	1064 nm	Pulse	1. Root canal treatment: Aids in the removal of harmful microorganisms and debris from the root canal system 2. Comprehensive periodontal surgery and scaling to remove dead tissue and harmful microorganisms 3. Removal of dental caries 4. Hemiglossectomy 5. Palliation of advanced neoplasms
Erbium:YAG	2940 nm	Pulse	1. Caries removal 2. Cavity preparation in enamel and dentin 3. Root canal preparation
Erbium, chromium-doped yttrium, scandium, gallium and garnet (Er,Cr:YSGG)	2780 nm	Pulse	1. Enamel etching 2. Caries removal 3. Cavity preparation 4. Bone ablation without over-heating, melting or changing the calcium and phosphorus ratios 5. Root canal preparation 6. Maxillary frenectomy 7. Lingual frenectomy 8. Exposure of unerupted teeth 9. Gingival fibroma excision 10. Pyogenic granuloma excision 11. Mucocele excision
Argon	572 nm	Pulse or continuous	1. Polymerization of restorative resin materials 2. Tooth bleaching 3. Elimination of necrotic tissue and gingival contouring/ Gingival hyperplastic lesions removal Treatment of oral lesions such as recurrent aphthous ulcers or herpetic 4. lesions 5. Frenectomy and gingivectomy 6. Oral mucous cysts 7. Decontamination of infected rough implant surfaces
Diode	810 or 980 nm	Pulse or continuous-wave	1. Proliferation of fibroblasts and enhancing the healing of oral lesions or surgical wounds 2. Gingivectomy, frenectomy, and gingivoplasty 3. Correcting the gingival contouring for esthetic purposes 4. Initial periodontal therapy 5.Implant therapy 6. Root surface modification 7. Osseous and soft tissue surgery 8. Gingival recontouring and depigmentation 9. Mucosal remodeling 10. Dental crown lengthening
Holmium:YAG (Ho:YAG)	2100 nm	Pulse	1. Gingival contouring 2. Treatment of oral lesions 3. Frenectomy and gingivectomy 4. Oropharyngeal squamous cell carcinoma

Various modalities of lasers and their extensive applications within the field of dentistry constitute a fundamental aspect of contemporary dental methodologies, emphasizing the advancement of therapeutic techniques. The lasers that are predominantly employed in dental practice include the Nd-YAG laser, erbium: yttrium aluminum garnet (Er: YAG) laser, carbon dioxide (CO2) laser, erbium chromium: yttrium scandium gallium garnet (Er-Cr:YSGG) laser, holmium:yttrium aluminum garnet (Ho:YAG) laser, in addition to diode lasers (Table-1, Figure-2).The ideal laser type for oral soft tissue surgeries remains inadequately documented. This study evaluated the effectiveness of Diode lasers (635 nm, 808 nm, 810 nm, and 980 nm), 5 on CO2 lasers (10,600 nm), 3 on Er, Cr: YSGG lasers (2780 nm), and 1 on KTP lasers (532 nm). All studies reported favorable clinical outcomes for mucocele excision, demonstrating improved intraoperative and postoperative metrics. The characteristics of various dental lasers and their applications of them summarized in Figure-1 and Table-1 [15][16]. These sophisticated devices are utilized for a plethora of soft-tissue and hard-tissue interventions performed within the oral cavity and adjacent orofacial structures, catering to a comprehensive range of treatments such as cavity preparation, caries identification, adhesive restoration, thorough cleaning of root canals, periodontal surgical procedures, management of peri-implantitis, and complex maxillofacial operations. The Nd: YAG laser, distinguished by its 1064 nm wavelength, operates in a pulsed manner and is particularly adept at the ablation and incision of soft-tissue lesions while concurrently facilitating the extraction of initial carious lesions [15][18].

Conversely, the CO2 laser, with a wavelength of 10,600 nm, can operate in either pulsed or continuous modes; however, its tissue penetration is confined to a mere 0.03-0.1 mm, rendering it most suitable for interventions involving soft-tissue incision, ablation, reepithelialization, and various periodontal surgical techniques [19].

The Er: YAG and Er-Cr: YSGG lasers, characterized by wavelengths of 2940 nm and 2780 nm respectively, are primarily utilized in endodontics, specifically for root canal preparation, extraction of carious material, and cavity design. Notably, the Er-Cr: YSGG laser possesses the distinctive ability to ablate bone without causing charring or altering the critical calcium-phosphorus balance. In current practice, the diode laser has gained notable popularity, composed of gallium-arsenide and operating at a wavelength of 904 nm; its primary application lies in soft-tissue procedures [20][21][22]. The diode laser represents a contemporary treatment option that was introduced into the field of dentistry in 1999. It is classified as a solid-state semiconductor, utilizing a blend of gallium (Ga), and arsenide (As), along with additional elements like aluminum (Al) and indium (In), and operates within a wavelength range of 810 to 980 nm [15][20]. Previous research has demonstrated the effectiveness of this technology in excising premalignant lesions associated with oral mucosal and maxillofacial conditions. Both high-powered (hot, hard) and low-powered (cold, soft) diode lasers are applicable for treating benign tumors, including irritation fibromas and fibrous epulides [23][24]. Additionally, diode lasers are particularly utilized in various aesthetic treatments, including gingival recontouring and depigmentation, periapical surgeries, operculectomies, and prosthetic surgical interventions such as mucosal remodeling in edentulous areas, dental crown lengthening, frenectomies, and vestibuloplasty. Additionally, diode lasers are advised for the excision of benign oral lesions, including ranulas, mucoceles, pyogenic granulomas, gingival hyperplastic lesions, fibrous hyperplasia, epulis fissuratum, and hemangiomas [24][25].

The argon laser is primarily utilized in the oral cavity for soft tissue applications. It produces a high-intensity blue light that facilitates the photopolymerization of dental materials, enhancing the physical characteristics of dental restorations. Its ability to promote tissue coagulation and healing renders it an essential instrument in oral surgery and periodontal therapies [26][27][28].

LLLT, low-level laser therapy, is an innovative technique that has emerged for use in fields such as medicine, dentistry, and physiotherapy. This method provides therapeutic benefits, including enhanced wound healing and pain alleviation for patients. It involves the application of laser light energy to living tissues, producing biosimulation effects without significantly increasing temperature. A key advantage of LLLT is its non-invasive nature, which helps minimize the occurrence of edema and inflammation. Helium neon or cold lasers are utilized in a continuous wave emission and non-contact mode to achieve the desired bio simulative effects. The benefits of soft lasers include improved wound healing, bone remodeling and repair, restoration of neural functions following injury, and the modulation of immune responses and pain signals. LLLT may serve as either a supplementary or primary method in oral surgical procedures, given its encouraging effects on postoperative wound healing and pain management [29].

The integration of laser technology into pediatric dentistry is particularly beneficial, as it mitigates fear and anxiety in younger patients, while simultaneously achieving greater acceptance from their guardians. When clinicians choose to implement lasers for surgical or pulp-related interventions, they frequently observe enhanced cooperation from children, thereby improving the overall efficacy of treatment outcomes.

Laser technology is employed for various purposes in pediatric dentistry, including caries prevention, early identification of dental conditions, cavity restoration, management of dental trauma, and the conduct of minor surgical procedures in pediatric patients, and it is anticipated to establish itself as the benchmark in pediatric dental practice shortly. A comprehensive analysis of the diverse applications of lasers in pedodontic practice is detailed in the ensuing sections.

## Soft Tissue Applications of Diverse Lasers

Gingivectomy, gingivoplasty, and frenectomy are the most popular procedures carried out using lasers. Compared with the use of a conventional scalpel, lasers can cut, ablate, and reshape the oral soft tissue more easily, with no or minimal bleeding and little pain as well as no or only a few sutures. Laser surgery occasionally requires no local anesthetic or only a topical anesthetic. The use of electrosurgery also facilitates easy tissue incision accompanied by a strong hemostatic effect. Compared with electrosurgery, lasers have a higher comfort level in patients, resulting in less operative and postoperative pain and fewer complications [30][31].

Lasers are utilized in aesthetic procedures such as the recontouring or reshaping of gingival tissues and crown lengthening. The employment of certain lasers allows for more precise and delicate control over the depth and extent of soft tissue ablation compared to traditional mechanical instruments. Notably, the Er: YAG laser is regarded as a safe and effective tool for managing periodontal soft tissues in aesthetic contexts, as it can accurately ablate soft tissues using a variety of fine contact tips, resulting in rapid and favorable wound healing due to minimal thermal alteration of the treated area. Another application for lasers in aesthetic treatments is depigmentation [31][32]. Lasers such as CO2, diode, and Nd: YAG are effective in addressing melanin pigmentation. However, in regions of thin gingival tissue, these lasers pose a risk of inducing gingival ulceration and recession due to their comparatively intense thermal or deeply penetrative properties. In such instances, the Er: YAG laser presents a safer and more effective option for melanin depigmentation. In a study involving thirty-six patients, aged 14 to 51 years, who required labial frenectomies, randomly assigned participants to receive either scalpel or diode laser treatment. Soft tissue metrics, including keratinized gingiva width (KGW), attached gingiva width (AGW), and attached gingival thickness (AGT), were documented before the surgical intervention, immediately post-surgery, one week later, and at one, three, and six months postoperatively [33]. Furthermore, functional complications and morbidity levels (including pain, swelling, and redness) were assessed during the initial postoperative week utilizing a visual analog scale (VAS). The findings indicated statistically significant improvements in KGW, AGW, and AGT post-surgery for both treatment groups; however, no significant differences were observed between these groups. The VAS results demonstrated that patients undergoing diode laser treatment experienced less discomfort and fewer functional complications compared to those who underwent scalpel surgery. According to the clinical data gathered, the application of this specific laser wavelength for periodontal surgical procedures is demonstrated to be both safe and efficacious. Abdelfattah M (2018) discusses Er: YAG lasers, which are classified as solid-state lasers with erbium-doped yttrium aluminum garnet (Er: Y3 Al5 O12) as their lasing medium. These lasers typically emit light at a wavelength of 2940 nm, situated within the infrared spectrum. In contrast to Nd: YAG lasers, the energy output from an Er: YAG laser exhibits a significant absorption by water. The Fotona Er: YAG laser was employed utilizing a long pulse with parameter settings of 85 mJ, 1.25 W, and a frequency of 15 Hz for 4 seconds. The laser beam was defocused to create a circular area of 3 mm in diameter, thereby limiting beam penetration to 2-4 µ/pulse while enhancing the area treated [34]. A postoperative evaluation was conducted 24 hours post-procedure, revealing an absence of discomfort, tooth sensitivity, pain, bleeding, or any other complications. The patient reported no disruption in their daily routine. A follow-up appointment was scheduled after one week, during which healing was observed to be unremarkable, with no postoperative complications, and no additional supportive therapy was necessary. The gingival tissue appeared pink, robust, and healthy, resembling a normal state. The outcomes were exceptionally favorable, resulting in a complete transformation of the gingival appearance. Aesthetic considerations have assumed significant relevance in contemporary dental practice [34].

Among the articles reviewed, 10 studies utilized diode lasers with varying wavelengths. Most studies indicated satisfactory postoperative healing with minimal or no scarring, absence of postoperative discomfort or pain, and no complications or recurrence in the treated lesions. Additionally, these studies reported reduced procedure times, enhanced surgical site visualization, and effective hemostasis. The follow-up periods across all studies ranged from 8 days to 1 year [35]. The previous review summarized the general characteristics and results, while the quality of the studies was evaluated according to CARE guidelines. Notable benefits of laser treatment highlighted in the studies included reduced or eliminated pain and bleeding, effective hemostasis, shorter operating times, lower analgesic requirements, and antibacterial properties. The findings confirm the efficacy of laser therapy in treating oral mucocele in pediatric patients. However, Chinta et al. documented a case of recurrence occurring 4 weeks post-diode laser treatment. To prevent further recurrences, a second excision was performed, and a thermoplasticized splint was utilized to deter nail biting and lip irritation from the incisors. No recurrence was noted during the 6-month follow-up [36]. Furthermore, Romeo et al. employed KTP, Er, Cr: YSGG, and diode lasers for mucocele removal, concluding that the diode laser provided superior bleeding control and cutting efficiency due to its higher affinity for hemoglobin [37].In Farshad Khosraviani et al., research in 2019, individuals aged 21 years or younger were classified as the pediatric group. The lasers utilized—Er: YAG (2940 nm), CO2 (10,600 nm), Er, Cr: YSGG (2780 nm), and diode lasers (650, 660, and 975 nm)—demonstrated effective clinical outcomes in procedures such as mucocele excision, frenectomy, gingival incision, and re-contouring, as well as the treatment of vascular malformations. Furthermore, the 660-nm diode laser proved to be a beneficial adjunctive treatment for halitosis and gingivitis associated with multi-bracket orthodontic appliances. The studies highlighted several advantages of laser use, including reduced pain and bleeding, effective hemostasis, shorter operation times, decreased need for analgesics, and antibacterial properties. As either a primary or supplementary tool, lasers can play a significant role in addressing both surgical and non-surgical pediatric oral soft tissue issues [38].

In four out of the 17 papers reviewed, the use of biostimulators or low-power lasers (with a power of ≤ 0.5 W, diode) was associated with the treatment of gingivitis, pain management, and the promotion of healing after the marsupialization of ranulas and mucoceles. Additionally, these methods were found to alleviate halitosis when applied under a photodynamic therapy protocol. The anti-inflammatory properties, along with the stimulation of healing cells such as fibroblasts and collagen production, can enhance tissue repair through laser radiation. The analgesic effects of low-power lasers may be attributed to their ability to modulate the inflammatory response and increase the pain perception threshold by reducing nerve impulse transmission. Furthermore, the antibacterial action of lasers plays a crucial role in addressing halitosis [39][40][41][42]. A patient exhibited gingival hypertrophy as a consequence of calcium channel blocker administration, characterized by edematous, finger-like projections of the interproximal papillae. This particular form of enlargement serves to distinguish this tissue response from the more substantial and fibrotic enlargements associated with phenytoin and cyclosporine. In an experimental study, the patient was subjected to anesthesia using 2% lidocaine supplemented with 1:100,000 epinephrine. A periosteal elevator was strategically inserted between the soft tissue and the tooth to safeguard the underlying hard tissue from potential damage induced by the CO2 laser wavelength [43][44]. Lasing was executed utilizing a CO2 laser in a continuous wave mode at 7 watts. The minimal interproximal bleeding observed was attributable to the larger caliber of capillaries present within the finger-like projections of the hyperplastic tissue, exceeding the diameter of the lasing beam. A biologic bandage (char layer) was applied over the surgical area, thereby obviating the necessity for a conventional periodontal dressing. In the context of laser gingivectomy, it was observed that postoperative pain levels were diminished relative to those experienced following traditional gingivectomy, a phenomenon likely linked to the thermal energy produced by the laser, which inhibits pain receptors, and the resultant coagulation that fosters a dry, isolated environment, thereby reducing the risk of infection to the surgical site [43][44].

High-power lasers (HPL) have found extensive application in conservative surgical practices and periodontal tissue management. The benefits of HPLs over traditional surgical methods for soft tissue procedures, as delineated in the scholarly literature, encompass disinfection, enhanced hemostatic capabilities that facilitate intraoperative visualization, decreased procedural duration, and minimized postoperative discomfort [45]. Recurrent drug-induced gingival overgrowth (DIGO) associated with various clinical conditions has been addressed using HPL. Their research highlighted the use of lasers as either an adjunctive or alternative strategy in periodontal and peri-implant therapies. Soft tissue surgery represents a primary indication for laser application. Lasers such as CO2, Nd: YAG, diode, Er: YAG, and Er,Cr:YAG are widely recognized as effective instruments for these interventions. Laser treatments surpass conventional mechanical methods in terms of efficient ablation, decontamination, and hemostasis, while also resulting in reduced surgical and postoperative pain during soft tissue management. Laser or laser-assisted pocket therapy is anticipated to evolve into a novel technical approach within the field of periodontics. Among these, the Er: YAG laser exhibits considerable potential for root surface debridement, including calculus elimination and decontamination. When considering laser applications for osseous surgery, CO2 and Nd:YAG lasers are deemed inappropriate due to their propensity for causing carbonization and degeneration of hard tissues [46]. At present, the Er:YAG laser is regarded as safe and effective for periodontal osseous surgery when utilized in conjunction with water irrigation. Hegde et al. (2014) noted that the laser gingivoplasty technique contributed to aesthetic enhancements. Gingivoplasty is employed to rectify gummy smiles by increasing crown lengths for either aesthetic or functional objectives. The surgical intervention aims to restore the biological width apically while facilitating greater exposure of dental structures. Historically, traditional surgical methodologies constituted the primary treatment options for soft tissue operations. The clinical crown height and gingival contour attained through laser therapy were notably impressive [47][48][49]. When hyperactive labial frenum is present, a laser-assisted frenectomy could be done with Er: YAG laser in an attempt for diastema closure.Er: YAG laser is also used for surgical management of severe tongue tie or ankyloglossia in infants and children. The CO2 laser is utilized in gingivectomy procedures and is additionally employed for the surgical excision of soft-tissue tumors within the oral cavity [50][51].

A series of cases involving seven children, aged between 6 and 14 years, were managed for various soft tissue conditions. These included crown lengthening, exposure of an unerupted molar, lingual and maxillary frenectomies, gingivectomy, excision of a pyogenic granuloma, and pulpotomy. All procedures were performed using an Er,Cr:YSGG laser for the effective removal of the relevant soft tissues. Each case demonstrated satisfactory healing, with follow-up conducted throughout 3 to 4 years [23].

In a study in 2010, various laser systems were employed, including a diode laser at 810 nm, an Er, Cr: YSGG laser at 2780 nm, and an Erbium:YAG laser at 2940 nm, for applications in soft tissue surgery, enamel etching, and biostimulation. Each wavelength was utilized with specific parameters tailored to the requirements of minimally invasive therapy, as supported by current international research. The outcomes from the cases documented indicated rapid and effective healing of the tissues treated with lasers. These procedures, essential for orthodontic treatment or its completion, are rendered straightforward, safe, and efficient, allowing orthodontic specialists to perform them independently. In conclusion, the laser technique demonstrates significant efficacy in various operative and surgical interventions during orthodontic therapy. Nonetheless, additional research is needed to establish standardized treatment protocols for orthodontic biostimulation [35].

Arora S (2016) notes that in periodontal surgical practices, crown lengthening entails the partial excision of supporting periodontal tissues to enhance the visibility of tooth structure. Various clinical scenarios necessitate crown lengthening, including aesthetically displeasing gingival heights, insufficient crown length, subgingival carious lesions, and crown fractures. For teeth exhibiting a sulcus depth exceeding 4mm on the facial aspect, gingivectomy may be indicated to achieve crown lengthening. Laser wavelengths ranging from 800 to 980 nm demonstrate minimal absorption in water but are significantly absorbed by other pigments. Due to its thermal effects, it generates a substantial coagulated layer, and the application of the diode laser has not been associated with adverse effects on the root surface. Therefore, it is posited that soft tissue laser surgery can be conducted with a high degree of safety concerning hard tissue [52]. Kutsch V asserts that lasers have emerged as invaluable clinical instruments within dental practice. The Er,Cr:YSGG laser serves as a multifunctional tool for crown-lengthening procedures, providing excellent hemostasis and effective recontouring of both gingival and osseous tissues. This approach is less invasive compared to conventional surgical techniques and facilitates the execution of the entire crown lengthening and crown preparation in a single appointment, yielding predictable clinical outcomes. Lasers are currently demonstrating remarkable efficacy across a diverse array of restorative and esthetic applications, including crown lengthening [53].

To facilitate the exposure of an unerupted or partially erupted tooth, the placement of an orthodontic bracket or button is typically accomplished through the utilization of laser technology. Given that the surgical field produced by laser techniques is characterized by an absence of significant bleeding, it becomes feasible to achieve the immediate placement of either brackets or buttons after the laser procedure is completed. Among the various types of lasers employed for this specific orthodontic application, the Erbium:Yttrium-Aluminum-Garnet (Er:YAG), Neodymium:Yttrium-Aluminum-Garnet (Nd:YAG), and Erbium-Chromium:Yttrium-Scandium-Gallium-Garnet (Er-Cr:YSGG) lasers are predominantly utilized to ensure optimal results in bracket or button placement [54][55].

In order to preserve the vitality of the dental pulp, lasers with differing wavelengths are strategically employed, operating at a power output that typically ranges between 0.5 to 1 watt. These specific lasers are activated in a pulsed mode, notably without the presence of water, and are utilized at a low frequency for a duration of approximately 10 seconds, a methodology that is specifically designed to significantly reduce the likelihood of coagulation occurring within the tissue. The CO2 laser, in particular, is indicated for performing pulpotomy procedures specifically in primary teeth, with the operational power settings being carefully calibrated between 1 and 4 watts, necessitating that the laser is employed in a non-continuous manner in order to avert any excessive thermal exposure that could potentially jeopardize the integrity of the underlying pulp tissue [56]. One of the notable drawbacks associated with the repeated application of lasers aimed at achieving complete excision of pulp tissue is the formation of a carbonized layer that can develop on the surface of the root canal; this layer, in order to ensure proper healing and functionality, must be meticulously removed through the irrigation process utilizing a solution comprised of 3% hydrogen peroxide and 5.25% sodium hypochlorite. In the year 1989, a significant study conducted by Ehihara revealed that the utilization of the Nd:YAG laser for pulpotomy procedures resulted in markedly improved wound healing in cases involving amputated pulp tissue. Furthermore, the diode laser, when implemented for pulpotomy in primary dentition, exhibited an impressive success rate of 100% after a one-year follow-up period, thereby establishing itself as a superior alternative compared to traditional methods involving ferric sulfate and electrosurgery, as evidenced by both clinical outcomes and radiographic assessments. In 1999, a comprehensive investigation conducted by Jeng-fen Liu and colleagues analyzed the outcomes associated with laser pulpotomy in primary teeth, revealing that all teeth treated with laser technology demonstrated clinical success during a follow-up visit conducted six months post-treatment, with the exception of just one isolated case [56][57][58].

The CO2 laser serves a pivotal role in the procedure of direct pulp capping, as it effectively manages hemorrhage and sterilizes the site of exposure, thereby facilitating the optimal placement of calcium hydroxide paste at the affected area and yielding favorable clinical outcomes. Typically, this laser irradiation is executed at a power setting ranging from 1 to 2 watts. The energy emitted by the laser possesses both an obtundant and sedative effect on inflamed pulpal tissue, additionally contributing to the closure of dentinal tubules, which is critical for maintaining pulp health. The mechanisms through which it aids in the process of indirect pulp capping are believed to be analogous to the sedative effects produced by laser treatment in cases of pulpitis [59][60].

Moreover, low-level laser therapy (LLLT) has been shown to facilitate the transformation of gingival fibroblasts into myofibroblasts, which in turn contributes significantly to the process of wound contraction. Remarkably, this beneficial effect can be observed as early as one day following the administration of laser treatment. The proliferation of fibroblasts is notably stimulated when a low dose of 2 joules per square centimeter is utilized, whereas at a considerably higher dose of 16 joules per square centimeter, fibroblast proliferation is suppressed, indicating a dose-dependent response to laser application. Given its properties that promote wound healing, LLLT has found extensive application in the therapeutic management of conditions such as recurrent aphthous ulcers, mucositis, and oral ulcers induced by radiation therapy, where it helps enhance recovery and alleviate discomfort. Historically, soft tissue surgical interventions in children were frequently avoided due to concerns about their uncooperative behavior, leading to the belief that such procedures could only be conducted under general anesthesia [61]. However, various studies have indicated that the application of lasers in both soft and hard tissue surgeries results in reduced discomfort and is generally well-received by young patients and their guardians. Consequently, the use of lasers can help alleviate psychological distress and anxiety associated with dental visits. This is exemplified in a case series involving six pediatric patients, where the Er,Cr:YSGG laser was effectively utilized for soft tissue surgeries [62][63][64]. Additionally, Sarkar et al. documented four cases involving different soft tissue lesions, including peripheral ossifying fibroma, traumatic fibroma, intraoral lipoma, and gingival fibroma. Notably, patients experienced no discomfort during or after the laser procedures, did not require local anesthesia, and no sutures or antibiotics were necessary. Observations included minimal bleeding, absence of edema, and favorable wound healing outcomes [65].

Laser Doppler flowmetry represents a noninvasive technique utilized for the assessment of pulp vitality. This method employs a semiconductor diode laser, utilizing helium, neon, and gallium aluminum, operating at a power level of 1-2 mW, to quantify the variations in red blood cell perfusion within the pulp tissue. During root canal treatment, the laser predominantly employed for disinfection is the pulsed Nd: YAG or Er: YAG laser, coupled with the irrigation of the canal utilizing 5.25% sodium hypochlorite or 14% ethylenediaminetetraacetic acid during the laser application. The bleaching gel used consists of peroxide, which, via oxidative pathways, produces a bleaching effect. The interaction of laser radiation with this bleaching agent generates thermal energy, consequently accelerating the process of oxidation [66][67].

Biolase technology is utilized in the fabrication of pediatric crowns. This apparatus is calibrated to deliver 5.5 W with a mixture of 65% air and 55% water, and the crowns are produced adhering to the same parameters as those employed in conventional techniques. This methodology eliminates the necessity for local anesthesia, thereby improving patient comfort. Furthermore, it enhances micromechanical bonding with resin cement, as it creates increased surface roughness on the treated dental substrates [68].

Various studies have concentrated on comparing conventional surgical techniques with carbon dioxide (CO2) laser treatment concerning outcomes in oral soft tissue pathology. The research assessed parameters including the need for anesthesia, levels of postoperative pain, and both intraoperative and postoperative complications. A total of 43 patients, of both genders, with a mean age of 54 years requiring soft tissue interventions, were randomly assigned to receive either traditional surgery using a scalpel or CO2 laser treatment. In the conventional surgical cohort, all participants were administered local anesthesia, whereas only 10 (42%) individuals in the laser cohort necessitated local anesthesia subsequent to the application of a topical anesthetic. Post-surgery, 18 (90%) patients in the conventional group required analgesics, in contrast to merely seven (29%) in the laser cohort. No complications were documented for either surgical method. Histological analysis of 39 specimens revealed that collateral thermal damage at the incision site did not alter the histopathological diagnosis. The results indicate that the CO2 laser serves as a highly effective instrument for excisional biopsies of soft tissues, associated with minimal complications and effective pain management. Consequently, CO2 laser methodologies are advocated as a feasible alternative to conventional surgical techniques for oral soft tissue interventions [35].

## Conclusion

The review indicates that laser therapy is a viable option for treating oral mucoceles and various soft tissue lesions in children. Research has shown that patients experience satisfactory healing post-operation, with minimal scarring and a notable decrease in pain and bleeding when compared to traditional surgical techniques. Specifically, diode laser treatments were linked to fewer functional complications and reduced discomfort during recovery. Patients who received laser treatment reported lower pain levels and required fewer pain relief medications than those who had conventional scalpel surgery. The results underscore multiple benefits of laser therapy, such as effective blood control, reduced surgery duration, and the removal of suture requirements. These advantages create a more positive clinical experience, particularly for pediatric patients who may feel more apprehensive about dental interventions.

## Conflict of Interest

None.
